# Reclassification of Hypophosphatemic Bone Disease as X-Linked Hypophosphatemia Following Genetic Testing in Adulthood

**DOI:** 10.1016/j.aed.2025.09.002

**Published:** 2025-09-11

**Authors:** Loay Kamel, Abdullah Hussain, Ahmed Elmewafy, Dalal S. Ali, Aliya A. Khan

**Affiliations:** 1 School of Medicine, Royal College of Surgeons in Ireland, Dublin, Ireland; 2 School of Medicine, Royal College of Surgeons in Ireland, Busaiteen, Bahrain; 3 School of Medicine, University of Sheffield, Sheffield, United Kingdom; 4 Division of Endocrinology and Metabolism, McMaster University, Hamilton, Ontario, Canada

**Keywords:** X-linked hypophosphatemia, XLH, genetic testing, PHEX gene, hypophosphatemia

## Abstract

**Background:**

X-linked hypophosphatemia (XLH) is a disorder caused by a pathogenic variant in the phosphate-regulating endopeptidase homolog X-linked gene. This leads to increased fibroblast growth factor 23 synthesis, prompting renal phosphate wasting and hypophosphatemia. Adults with XLH present with osteomalacia, chronic musculoskeletal pain, enthesopathy, osteoarthritis, fractures, and pseudofractures. We present a patient initially diagnosed with hypophosphatemic, nonrachitic bone disease. However, later genetic testing identified a pathogenic phosphate-regulating endopeptidase homolog X-linked variant (c.1645+1G>A), establishing an XLH diagnosis.

**Case Report:**

A 50-year-old male was diagnosed with XLH through molecular testing at age 49. He was initially diagnosed at age 3 years with hypophosphatemic bone disease due to an unclear inheritance pattern. Treatment with phosphate and active vitamin D was started but discontinued at age 13 due to adverse effects, then resumed between ages 30 and 45. The patient presented with joint pain, abnormal gait, dental abscesses, and right hip replacement due to early-onset osteoarthritis with history of an atraumatic vertebral fracture at age 42. Labs showed hypophosphatemia, low tubular phosphate reabsorption, and elevated alkaline phosphatase and fibroblast growth factor 23. He was switched to burosumab therapy, resulting in clinical improvement.

**Discussion:**

This case underscores the diagnostic challenges of atypical XLH, particularly when X-linked inheritance patterns are absent. It illustrates the transformative role of molecular diagnostics in establishing diagnoses. It also reports on the impact of burosumab therapy when initiated in adults.

**Conclusion:**

This case demonstrates hallmark biochemical and radiological features of XLH, and the value of genetic testing in establishing a definitive diagnosis, particularly in individuals with atypical or nonclassical presentations.

**Highlights**

• Genetic testing is crucial for accurate diagnosis of X-linked hypophosphatemia (XLH), especially in cases with atypical presentations or unclear inheritance patterns
• Early recognition of XLH is key to minimizing skeletal, dental, and soft tissue complications
• This case highlights the evolution of diagnostic approaches in XLH, emphasizing the transition from reliance on clinical and biochemical assessments to the incorporation of molecular genetic testing for definitive diagnosis

**Clinical Relevance**

This case underscores the utility of genetic testing in reclassifying atypical presentations of hypophosphatemic bone disease as X-linked hypophosphatemia. It highlights its essential role in resolving longstanding diagnostic uncertainty and guiding appropriate management.

## Introduction

X-linked hypophosphatemia (XLH) is a rare hereditary disorder characterized by impaired phosphate homeostasis due to pathogenic variants in the phosphate-regulating endopeptidase homolog X-linked (*PHEX*) gene.^1^ These variants lead to excessive fibroblast growth factor 23 (FGF23) activity, resulting in increased renal phosphate excretion, chronic hypophosphatemia, and defective skeletal mineralization.^1^ XLH primarily manifests in childhood with rickets, short stature, lower limb deformities, and dental abscesses.^1^ In adults, complications such as osteomalacia, enthesopathy, early-onset osteoarthritis, fractures, and pseudofractures are common.^1^

The diagnosis of XLH is established in the presence of persistent hypophosphatemia, low tubular phosphate reabsorption relative to glomerular filtration rate (TmP/GFR), and, in most cases, elevated or inappropriately normal FGF23 levels.^2^ Radiographic findings in XLH vary with age and disease progression. In children, radiographs commonly show features of rickets, such as cupping, fraying, and widening of the metaphyses, particularly at the wrists and knees.^3^ Bowing of the long bones, particularly the femur and tibia, is also frequently observed. As patients transition into adulthood, radiographic features shift towards enthesopathy, osteomalacia, and early-onset osteoarthritis, particularly in weight-bearing joints.^4^ Adults may also develop pseudofractures, sclerosis of the long bones, and spinal osteophytes. In some cases, mild diaphyseal sclerosis—such as in the femur—can be seen, which may be subtle and nonspecific without a clear history.^5^ These findings not only support diagnosis but also help assess the burden of skeletal complications and the need for intervention.

Typically, inheritance follows an X-linked dominant pattern, with a family history of the condition being a strong indicator of the diagnosis. Genetic confirmation of pathogenic or likely pathogenic *PHEX* variants has improved diagnostic accuracy, particularly in atypical cases.

Before the discovery of *PHEX* and the availability of genetic testing in the late 1990s, several cases of hypophosphatemia were misclassified as "hypophosphatemic bone disease" (HBD). Without genetic confirmation, clinicians relied on biochemical markers and family history, leading to uncertainty in distinguishing XLH from other inherited and sporadic causes of hypophosphatemia.

In some cases—including this patient’s—the condition was initially believed to follow an autosomal inheritance pattern rather than X-linked transmission, which contributed to prolonged diagnostic uncertainty. The patient described in our study was first reported by Scriver et al in the American Journal of Medical Genetics in 1977, where the condition was classified as HBD due to its atypical presentation and unclear inheritance pattern.^6^ Although it was recognized that he had a genetically transmitted form of hypophosphatemia, the underlying pathology and mode of inheritance remained unknown at the time. The condition was presumed to have been inherited paternally, as a cousin on his father’s side had been diagnosed with hypophosphatemic rickets.

Historically, treatment of XLH—and of cases misclassified as HBD—consisted of phosphate supplements and active vitamin D analogs (calcitriol or alfacalcidol). However, this regimen was suboptimal, due to gastrointestinal side effects, inconsistent efficacy, and long-term complications, including nephrocalcinosis and secondary hyperparathyroidism. In cases similar to our patient where the diagnosis remained uncertain for decades, prolonged inadequate treatment contributed to skeletal complications, including early fragility fractures, joint destruction, and stiffness as well as reduced physical function, chronic pain, fatigue and impaired quality of life. The introduction of genetic testing for *PHEX* variants in 1999 significantly improved diagnostic accuracy, reducing misdiagnosis and misclassification of XLH as HBD.

The introduction of burosumab, a monoclonal antibody targeting FGF23, has transformed XLH management. By inhibiting FGF23, burosumab restores phosphate homeostasis, improving skeletal mineralization, reducing bone pain, healing fractures, and pseudofractures. It is now recommended as the first-line treatment for children. Burosumab is also strongly recommended in adults who have experienced fractures or pseudofractures, as compared to receiving no treatment.^1^^,^^2^

## Case Report

### Presentation

A 50-year-old male, presented to our tertiary referral center with a lifelong history of hypophosphatemia, progressive polyarthralgia, bone pain, and a history of multiple atraumatic vertebral fractures. He reported worsening joint pain, exercise intolerance, and a recent hip replacement surgery due to osteoarthritis. He also had a history of dental abscesses and ectopic calcifications. He experienced multiple musculoskeletal symptoms over time, including chronic joint pain, muscle weakness, reduced mobility, enthesopathy, early-onset osteoarthritis, and atraumatic vertebral fractures (T12 and L1). Other medical comorbidities include sleep apnea on CPAP, hypertension (diagnosed at the age of 39 years), fatty liver (identified on liver ultrasound at the age of 46 years), allergic rhinitis, dyslipidemia, and depression (after quitting his job due to disability).

### Family History

Several family members show features consistent with hypophosphatemia. On the maternal side, the patient’s grandfather was reportedly diagnosed with hypophosphatemia and advised to drink 6 cans of Coca-Cola per day; he had no teeth, though fracture history is unknown. His maternal uncle had frontal bossing, short lower limbs, and dentures, but no reported bowing or fractures. On the paternal side, a cousin was diagnosed with hypophosphatemia—his mother was also affected. The patient’s mother is unaffected. Among the patient’s siblings and children, a half-brother is unaffected, and all 3 biological children have normal phosphate levels.

### Examination

On physical examination, the blood pressure was 134/97 mmHg. His standing height was (5'6.5"), weight was 90.7 kg, and a body mass index of 31.8 kg/m^2^. He had an abnormal (waddling) gait, and mild bowing of the lower limbs. He had a slight frontal bossing of skull and a mild lumbar lordosis. There was a notable polyarticular tenderness and reduced range of motion in multiple joints. Dermatological findings included recurrent skin lesions on the fingers. Head and neck, cardiovascular, and respiratory examination was unremarkable.

### Investigation

Laboratory tests revealed persistent hypophosphatemia-serum phosphorus, low TmP/GFR, mildly elevated alkaline phosphatase, normal serum corrected calcium, 1,25-dihydroxyvitamin D, 24-hour urinary creatinine, 24-hour urinary calcium, PTH, and 25 hydroxyvitamin D levels (Table). Genetic testing identified a pathogenic splice donor variant in the *PHEX* gene (c.1645+1G>A), with findings suggestive of somatic mosaicism. This confirmed the diagnosis of XLH. Skeletal survey showed mild femoral sclerosis without significant fractures, anterior osteophytes at L3-L4, and renal ultrasound ruled out nephrocalcinosis.

**Table:** Laboratory Values for the Patient at the Time of Admission

Laboratory test	Value	Units	Normal range
PO_4_	0.60	mmol/L	1.12-1.45
TmP/GFR	0.493	N/A	0.9-1.35 (for males)
ALP	127	IU/L	30-120
Ionized calcium	1.26	mmol/L	1.15-1.33
Total calcium	2.38	mmol/L	2.15-2.55
1,25 dihydroxyvitamin D	109	pmol/L	75-200
24-h urinary creatinine	11.0	mmol/d	7.8-20
24-h urinary calcium	5.42	mmol/d	2.5-7.5
25 hydroxyvitamin D	67	nmol/L	50-125

Abbreviations: ALP = alkaline phosphatase; PO_4_ = phosphate; TmP/GFR = tubular maximum reabsorption of phosphate per glomerular filtration rate.

### Differential Diagnosis

The differential diagnosis includes XLH, autosomal dominant hypophosphatemic rickets, and tumor-induced osteomalacia.

### Management

Initially, the patient was treated with oral phosphate and calcitriol during childhood; however, therapy was discontinued due to gastrointestinal intolerance. In January 2022 (at age 49 years), he was initiated on burosumab therapy (starting at 90 mg subcutaneously monthly, later adjusted to 80 mg monthly). This led to normalization of serum phosphorus levels, (TmP/GFR improvement) and a substantial improvement in musculoskeletal symptoms and physical health. The patient no longer requires regular use of analgesics or intra-articular corticosteroid injections, which had been prescribed for him for over 15 years. The patient has reported an approximate 80% improvement in mobility and quality of life since initiating burosumab therapy 3 years ago to date. He developed left shoulder pain with reduced range of motion; imaging showed no fracture, and frozen shoulder was diagnosed, for which he received an intra-articular steroid injection. In his latest follow-up, he did not report muscle cramps, fractures, or new dental problems.

#### Lifetime Skeletal and Musculoskeletal Complications

In this case, lifetime skeletal and musculoskeletal complications included lower limb bowing deformities (bilateral femurs and tibiae), short stature (below the third percentile), and osteomalacia secondary to chronic hypophosphatemia. The patient also experienced atraumatic vertebral fractures, specifically grade 2 anterior wedge compression fractures at T12 and L1, identified on spine X-rays at age 42 (likely remote), and early-onset osteoarthritis, severe enough to necessitate a right total hip replacement at age 47. Additional complications comprised chronic joint pain, enthesopathy (pain and stiffness at tendon/ligament insertions), limited range of motion in multiple joints, and persistently low bone mineral density, particularly at the radial site (cortical bone).

#### Dental Complications

Dental complications in this case included multiple dental abscesses since childhood, early tooth loss with at least 2 molars lost in adulthood, and frequent cavities.

#### Soft Tissue Complications

Soft tissue complications included ectopic calcifications, manifesting as painful subcutaneous nodules, particularly on the fingers and bilateral shoulder bursitis, which was managed with corticosteroid injections.

## Discussion

This study underlines many key findings pertaining to XLH including atypical presentation, genetic testing, associated complications and treatment options. The presenting patient exhibited an unusual inheritance pattern, as his mother was unaffected, which is inconsistent with the X-linked dominant transmission typically seen in XLH. In classic XLH, the condition is passed from affected mothers to their children, and males do not transmit the condition to their sons. The absence of maternal involvement led clinicians to consider alternative diagnoses. At the time, the lack of access to genetic testing further contributed to diagnostic uncertainty, and the patient was diagnosed with HBD, a descriptive label used before specific genetic mutations could be identified. Although he was managed in a similar manner to XLH management in the past, the delay in confirming XLH diagnosis, has delayed his transition to burosumab, which resulted in a significant clinical and biochemical improvement. The severity of his condition and discontinuation of conventional therapy during adolescence due to adverse events has led to numerous complications including dental abscesses, osteomalacia, vertebral fractures, bone deformities, and early-onset osteoarthritis. Advancements in genetic testing allowed healthcare professionals to find variants in the *PHEX* gene, eventually leading to a definitive diagnosis of XLH. The patient was then started on burosumab therapy at the age of 49 years, which stabilized his phosphate levels and improved his quality of life substantially.

This case highlights the critical role of genetic testing in the diagnosis of XLH, particularly in atypical presentations where the mode of inheritance is unclear, such as in this patient. At the time, Scriver et al were unable to establish a definitive diagnosis, as the inheritance pattern was initially believed to be autosomal. Contributing factors to the delayed diagnosis included the atypical presentation and the unavailability of genetic testing.

Our findings reinforce the importance of considering XLH in patients with unexplained hypophosphatemia secondary to renal phosphate wasting, and skeletal symptoms, even in the absence of classic X-linked inheritance pattern. Furthermore, our support the growing body of literature demonstrating the efficacy of burosumab in adults. Studies such as Insogna et al (2019) and Imel et al (2020) have shown similar improvements in musculoskeletal symptoms and quality of life following burosumab therapy in adults.^5^^,^^7^ This case also underlines the need for routine genetic screening in patients with diagnostic uncertainty. Early identification enables targeted treatment, reduces complications, and improves long-term outcomes and quality of life.

The reclassification of the case originally reported by Scriver et al aligns with findings from more recent genetic studies that highlight the diagnostic limitations of earlier clinical evaluations.^6^ Fahiminiya et al demonstrated that clinical and biochemical presentations alone may lead to misdiagnosis, particularly in phenotypically complex disorders. In their study, whole exome sequencing (WES) revealed a de novo splicing mutation (c.1645+1G>A) in *PHEX* in a female proband who had initially been diagnosed with autosomal recessive hypophosphatemic rickets.^8^ This genetic finding led to a revision of the clinical diagnosis of XLH, underscoring the value of molecular diagnostics in clarifying cases where traditional clinical assessments had been inconclusive or misleading.

Gaucher et al support the central role of *PHEX* variants in the majority of XLH cases. In their analysis of 118 pedigrees, *PHEX* mutations were found in 87% of familial cases and 72% of sporadic cases.^9^ Their findings also highlight that de novo mutations can occur, as evidenced by their identification of *PHEX* variants in sporadic cases where parents were unaffected.^9^ This provides a potential explanation for misinterpretations of inheritance patterns in the case reported by Striver et al. Moreover, they emphasize the necessity of complete sequencing of all 22 exons of the *PHEX* gene when missense variants are involved, due to their frequent clustering in key protein regions.

Ichikawa et al also identified a wide spectrum of *PHEX* variants in patients with XLH, including 13 novel variants and, notably, a variant in the 3’-untranslated region (c.∗231A>G), which was not previously associated with the disorder.^10^ This variant was found in eight patients from seven kindreds, none of whom had other detectable variants in the *PHEX* coding region. It was not found in over 800 normal chromosomes, suggesting its pathogenic relevance. Their findings reinforce the conclusion that no single *PHEX* variant predominates in XLH and that variants may occur across a wide range of genomic regions, including noncoding sequences, further complicating clinical diagnosis in the absence of genetic testing.

Collectively, these studies demonstrate that molecular genetic testing, especially WES and full *PHEX* gene sequencing, can provide definitive diagnostic clarity, particularly in patients whose clinical presentation does not fully conform to established phenotypes. This directly supports the reinterpretation of the case described by Scriver et al, suggesting that cases previously thought to represent novel or undefined disorders may, in fact, be attributable to known genetic conditions such as XLH, once appropriate genetic analyses are applied.

## Conclusion

This case highlights an atypical presentation of XLH, initially misclassified as HBD due to the absence of a clear X-linked dominant inheritance pattern and milder early skeletal findings. The diagnosis remained uncertain for decades, underscoring the limitations of relying solely on clinical and biochemical features in the pre-genetic testing era. Genetic confirmation of a *PHEX* splice site variant ultimately clarified the diagnosis. The patient’s clinical improvement following burosumab initiation demonstrates the potential for meaningful benefit even in long-standing, previously untreated adult XLH. This case reinforces the importance of considering XLH in adults with unexplained hypophosphatemia and skeletal complications, regardless of family history, and highlights the critical role of genetic testing in resolving diagnostic uncertainty.

## Disclosure

The authors have no conflicts of interest to disclose.
